# Epidemiological study of Klebsiella pneumonia isolates producing KPC-2 carbapenemase in a general hospital over a four year period in Greece

**DOI:** 10.1186/2047-2994-4-S1-P131

**Published:** 2015-06-16

**Authors:** A Tatsiopoulos, K Tryfinopoulou, A Stamatiou, P Giakouppi, E Petinaki

**Affiliations:** 1 Microbiology, Leivadia General Hospital, Levadia, Greece; 2 Hospital acquired infections , Central Public Health Laboratory, Athens, Greece; 3 Internal Medicine, Leivadia General Hospital, Levadia, Greece; 4 Médecin Interne, Hôpitaux Universitaires de Genève, Geneva, Switzerland; 5 Microbiology, National School of Public Health, Athens, Greece; 6 Microbiology, Larisa University Hospital, Larisa, Greece

## Introduction

In Greece *Klebsiella pneumoniae* producing KPC-2 carbapenemase (Kp-KPC+) constitute an important public health problem.

## Objectives

To investigate the epidemiology of Kp-KPC+ isolates in a secondary general hospital in Greece, with no prior history of Kp-KPC+ until 2010.

## Methods

Between 2011 and 2014,156 *Klebsiella pneumoniae* isolates were recorded from routine laboratory tests. Carbapenemases production was tested by Modified Hodge Test. Phenylboronic acid, EDTA or both along with meropenem discs were used as inhibitors for detection of *Klebsiella pneumoniae* carbapenemases (KPCs), metallo-beta lactamases (MBLs) or both carbapenemases, respectively. Susceptibility testing for various antimicrobial agents including carbapenems was performed by the disk diffusion method according to CLSI criteria. MICs were determined by Vitek-2 or E-test methodology. bla_KPC_, bla_VIM_ and bla_NDM_ genes were detected by PCR. Clinical data, related to admission, previous hospitalizations and outcome, were collected using patients’ medical records.

## Results

During the study period, 18 *Klebsiella pneumoniae* isolates were found as KPC-2 producers (11,5%). No other carbapenemase gene was detected in our hospital during the study period. The majority of Kp-KPC(+) isolates were recovered from urine cultures. All isolates were highly resistant to carbapenems, whereas susceptibility rates for colistin, tigecyclin and gentamicin were 100%, 92% and 77%, respectively. The first KPC-2 positive isolate was detected in September 2011. The yearly distribution of these patients was: 2011(3), 2012 (7), 2013 (4), 2014 (4). 10 patients had a history of previous hospitalization in tertiary care hospitals in Athens. Crude mortality rate among patients with Kp-KPC(+) was high (6/18, 33,3%). Colonization/infection during hospitalization was not identified.

## Conclusion

In our hospital there is a low prevalence of Kp-KPC+ compared to tertiary care hospitals in Athens. Given the antimicrobial therapy toxicity, the prolongation of hospitalization and the mortality due to possible therapy failure, special care should be taken.

## Disclosure of interest

A. Tatsiopoulos Consultant for: Microbiology, K. Tryfinopoulou: None declared, A. Stamatiou: None declared, P. Giakouppi: None declared, E. Petinaki: None declared.

